# Phenological mismatch drives selection on elevation, but not on slope, of breeding time plasticity in a wild songbird

**DOI:** 10.1111/evo.13660

**Published:** 2018-12-21

**Authors:** Jip J. C. Ramakers, Phillip Gienapp, Marcel E. Visser

**Affiliations:** ^1^ Department of Animal Ecology Netherlands Institute of Ecology (NIOO‐KNAW) 6700 AB Wageningen The Netherlands

**Keywords:** Fitness, genetic variation, life‐history evolution, phenotypic plasticity, quantitative genetics, natural selection

## Abstract

Phenotypic plasticity is an important mechanism for populations to respond to fluctuating environments, yet may be insufficient to adapt to a directionally changing environment. To study whether plasticity can evolve under current climate change, we quantified selection and genetic variation in both the elevation (RN_E_) and slope (RN_S_) of the breeding time reaction norm in a long‐term (1973–2016) study population of great tits (*Parus major*). The optimal RN_E_ (the caterpillar biomass peak date regressed against the temperature used as cue by great tits) changed over time, whereas the optimal RN_S_ did not. Concordantly, we found strong directional selection on RN_E_, but not RN_S_, of egg‐laying date in the second third of the study period; this selection subsequently waned, potentially due to increased between‐year variability in optimal laying dates. We found individual and additive genetic variation in RN_E_ but, contrary to previous studies on our population, not in RN_S_. The predicted and observed evolutionary change in RN_E_ was, however, marginal, due to low heritability and the sex limitation of laying date. We conclude that adaptation to climate change can only occur via micro‐evolution of RN_E,_ but this will necessarily be slow and potentially hampered by increased variability in phenotypic optima.

Phenotypic plasticity is an important mechanism by which an individual can adapt its phenotype in response to fluctuating environmental conditions (Pigliucci 2001; Schlichting and Pigliucci 1998). For example, life‐history traits such as phenology (e.g., timing of breeding or migration) or litter size are often phenotypically plastic, and this plasticity is adaptive as it tracks the environmental variability in the optimal phenotype (Scheiner 1993). Timing of avian seasonal reproduction (or laying date) is an illustrative example in this respect; since laying date is an important determinant of reproductive success and its optimal timing varies from year to year (Visser and Both 2005; Verhulst and Nilsson 2008), it is often phenotypically plastic to environmental conditions, usually spring temperatures (Brommer et al. 2005; Nussey et al. 2005b; Charmantier et al. 2008; Avilés et al. 2014). Similarly, avian clutch size can be phenotypically plastic with respect to population density, such that birds maximize the number of successfully raised, high‐quality offspring under varying levels of food availability and competition (Ricklefs 1980; Both et al. 2000; Sæther et al. 2016). More generally, many forms of animal behavior are highly context dependent in a wide range of taxa (Dingemanse et al. 2010), making phenotypic plasticity ubiquitous in nature (Pigliucci 2001).

Phenotypic plasticity can be described by a reaction norm (Scheiner 1993; Woltereck 1909); that is, the (often assumed to be linear) function of the phenotype against the environment, characterized by the intercept or elevation (i.e., the phenotype in the average environment) and slope (i.e., the sensitivity of the phenotype to the environment). The degree of plasticity may vary among individuals (individual‐by‐environment interaction or I × E) and this variance may have a partly genetic basis (genotype‐by‐environment interaction or G × E), making phenotypic plasticity itself an evolvable trait (Scheiner 1993; Van Tienderen and Koelewijn 1994; Via et al. 1995). In a directionally changing environment, evolution of the reaction norm may be necessary because the environmental driver of the trait no longer accurately predicts future environmental conditions, rendering plasticity alone insufficient to respond to environmental change (Visser 2008). Quantifying variation in reaction norms is therefore imperative for understanding evolutionary processes because it can elucidate whether populations are capable of responding to such directional selection. Predicting such responses may be difficult when G × E leads to nonlinear changes in genetic variation across environments (Tomkins et al. 2004; Turelli and Barton 2004; Kokko and Heubel 2008), or when genetic variation and selection are negatively correlated with one another (Wood and Brodie III 2016; but see Ramakers et al. 2018). Ultimately, the extent to which phenotypic plasticity modulates evolutionary processes will be highly context dependent (Hoffman and Merilä 1999).

A largely unexplored aspect in the light of directional environmental change is how selection on consumer phenology translates to selection on the reaction norm (Visser 2008). If the optimal reaction norm—that is, the relationship between resource phenology and the environment driving phenology of the consumer (the “cue”)—does not change over time, selection on neither the elevation nor the slope of the consumer reaction norm should occur. If, on the other hand, the sensitivity of the optimal reaction norm to the environmental cues changes over time, this may lead to two scenarios: selection for increased phenotypic plasticity to track the plasticity of the resource (Nussey et al. 2007; Lande 2009; Gienapp et al. 2014), or selection for reduced plasticity when the cue environment changes to such an extent that the consumer can no longer accurately predict the resource phenology (De Jong 1999; Reed et al. 2010). Alternatively still, if the elevation of the optimal reaction norm changes over time, selection on the elevation, rather than the slope, of the consumer reaction norm should occur. Global climate warming has led to the disruption of phenological synchrony between trophic levels (Visser and Holleman 2001; Both et al. 2006; Durant et al. 2007; Both et al. 2009; Schultz et al. 2009; Thackeray et al. 2010; Thackeray et al. 2016; Visser and Gienapp, unpubl. ms.) and in some cases to directional selection on consumer phenology (Van Noordwijk et al. 1995; Visser et al. 1998). It is unclear whether selection on phenology reflects selection on the elevation, the slope or both components of the reaction norm. Most of what we know about the evolutionary dynamics of the reaction norms stems from theoretical work or laboratory experiments (see Scheiner 1993; De Jong 1999; Van Asch et al. 2007; Lande 2009) or from phylogenetic and population comparisons of reaction norms (Murren et al. 2014), with very few empirical examples of how selection as well as (additive) genetic variation led to an evolutionary change in the reaction norm in wild populations (cf. Carter et al. 2017; Van Asch et al. 2013).

Several long‐term, vertebrate study populations have been shown to exhibit phenotypic plasticity in phenology (e.g., Réale et al. 2003; Brommer et al. 2005; Nussey et al. 2005a,b; Charmantier et al. 2008). Evidence for I × E and G × E, however, is overall mixed. For example, phenotypic plasticity of laying date against spring temperature in Dutch great tits (*Parus major*) was shown to vary between individuals (I × E), with part of this variation being heritable (G × E; Nussey et al. 2005b). In a UK great tit population, on the other hand, there was no I × E (Charmantier et al. 2008). In a reanalysis for both populations, phenotypic, but not genetic, variation was found to be present in the elevation as well as the slope of the reaction norm in both populations (Husby et al. 2010).

In general, heritable variation in phenological traits is widespread (e.g., van Noordwijk et al. 1981; Blondel et al. 1990; Réale et al. 2003; Sheldon et al. 2003), which in the absence of variation in plasticity slopes should reflect variation in the elevation of the reaction norm. Evidence for G × E in wild populations is, however, rare (Wood and Brodie III 2016; Hayward et al. 2018; but see Ramakers et al. 2018), and estimates of selection on reaction norms are even scarcer. This is because it requires estimating selection on both the elevation and the slope, which is a statistically challenging procedure (see discussion in Weis and Gorman (1990) and in Brommer et al. (2012)). Several studies used random regression techniques to get individual estimates (best linear unbiased predictions, or BLUPs) for reaction norm elevation and slope and performed a separate analysis on these components to quantify selection on them (e.g., Brommer et al. 2005; Nussey et al. 2005b). Such a two‐step approach is now considered inappropriate (Hadfield et al. 2010; Morrissey and Liefting 2016) and an alternative method has been suggested to use random regression models to estimate variation in reaction norms as well as selection thereon in a single analysis (Brommer et al. 2012). Application of this method to estimate selection on reaction norms in general has been rare (Brommer et al. 2012; Hayward et al. 2014). Thus, quantitative estimates of both (genetic) variation in and selection on reaction norms in wild populations are rare or ambiguous, and there is a clear need to empirically quantify the evolutionary dynamics of reaction norms in the light of environmental change (Visser 2008).

To address this gap, we quantified selection on and predicted evolution of the reaction norm of timing of breeding (laying date) in response to temperature (an important environmental cue; Schaper et al. 2012; Visser et al. 2009) in a Dutch long‐term (1973–2016) study population of the great tit (*Parus major*) at the Hoge Veluwe. Laying date is a labile trait that can be expressed several times by an individual; this means that each female has her own reaction norm. Timing of breeding is under increased directional selection (for earlier dates) in this population due to a climate change‐driven mismatch with the caterpillar food peak (Visser et al. 1998; Reed et al. 2016). We tested (i) whether the optimal (linear) reaction norm—determined by regressing the caterpillar biomass peak date against the temperature used by great tits to time laying date—changed over time, both in its elevation and slope; (ii) whether a change in the optimal reaction norm over time led to selection on the phenotypic reaction norm of the consumer (great tit); and (iii) whether there was genetic variation in great tit reaction norms. Based on the results, we used a quantitative genetic model to predict quantitatively the amount of expected change in the population reaction norm (elevation and slope) due to years of selection, and verified the outcome by comparing the predicted reaction norms with those observed in the wild. Combined, these results should elucidate whether the breeding‐time reaction norm can evolve given sustained directional selection.

## Methods

### DATA COLLECTION AND PREPARATION

Data were collected from a long‐term great tit (*Parus major*) population at the Hoge Veluwe National Park (HV; 52°23′N, 05°51′E, central Netherlands). The HV population is situated within a matrix of natural habitat, facilitating dispersal from and into the study area, and has been monitored continuously since 1955. Nest boxes are provided in excess (∼400) in suitable habitat. Each breeding season from April to July, boxes are checked at least once a week to monitor the breeding activity of hole‐breeding passerines. Clutch size is noted and laying date (i.e., the date when a female's first egg of her first clutch in that season is laid) is calculated based on the number of eggs in the nest, assuming that one egg is laid per day. During the chick‐feeding phase, parents and chicks are captured at the nest box and ringed, allowing for establishing a “social” pedigree. Extra‐pair paternity in the neighboring population of Westerheide ranges from 6.5% to 12.5% of all chicks (Van Oers et al. 2008), a common rate for tit species (Brommer et al. 2010) that has been found to only marginally affect the accuracy of heritability estimates when sample sizes are sufficiently large (Charmantier and Réale 2005; Firth et al. 2015).

Temperature data were retrieved as daily averages from a nearby weather station of the Royal Dutch Meteorological Institute (KNMI; Deelen station: 52°05′N, 5°87′E; http://projects.knmi.nl/klimatologie/daggegevens/). Temperature data were averaged over the period from March 11 to April 20, which is the time window yielding the strongest correlation between annual mean daily average temperature and annual mean laying date (*r* ^2^ = 0.74). This was done using a sliding‐window approach in the “climwin” R package (Bailey and Van de Pol 2017); details of the analyses are given elsewhere (Bailey et al. in prep.). We used these mean daily average temperatures as proxies for the environmental cues great tits use to time their reproduction, which in reality is an intricate interplay between day length and changes in temperature over time (Gienapp et al. 2005; Schaper et al. 2012).

The peak date of food availability has been estimated since 1985, using frass samples (see Visser et al. 2006 for details). The most common caterpillars are of the winter moth (*Operophtera brumata*) and the oak leaf roller (*Tortrix viridana*), although other species are present. Caterpillar peak date correlates very well with the mean temperature from March 22 to May 16 (*r* ^2^ = 0.81, resulting from a sliding window analysis); we used this relationship to hindcast the caterpillar peak date from 1973 to 1984 (all temperatures fell within the analyzed range).

Here, we consider brood data from 1973 to 2016, as the study area was reorganised following a major storm in 1972, and the latest data on recruitment of fledged offspring (our fitness measure) was available for 2017. During the study period, a number of broods were manipulated in brood size. None of the broods that we considered were affected in their laying date, but brood size manipulations could affect the reproductive success of a brood. We therefore included broods whose size was manipulated in the laying date analyses, but removed them from annual selection analysis (but not for analysis of selection on the reaction norm; see below). Sample sizes per analysis are given in Table 1.

**Table 1:** Sample sizes used in all analyses

Analysis	Year span	*N*_years_	*N*_females_	*N*_broods_
1. Optimal laying date vs. environment	1973–1987	15	—	—
	1988–2001	14	—	—
	2002–2016	15	—	—
2. Selection on plasticity^a^	1969–1987	19	456	1272
	1988–2001	14	365	953
	2002–2012	11	280	691
3. Quantifying ***G***‐matrix	1973–2016	44	3028	4890
4. Selection ($\beta_{z}$) on laying date^b^	1973–2016	44	>2347	3662
5. Observed reaction norms	1973–1987	15	1026	1650
	1988–2001	14	993	1551
	2002–2016	15	1126	1689

^a^Year span here indicates cohort span; birds with incomplete lifetime reproductive success (LRS) at the end of the dataset were omitted, whereas LRS of birds breeding in 1973 were complemented with brood data from previous years, hence making the cohort span 1969–2012 (see text). Only birds with ≥ 2 breeding events were included here.

^b^Reduced dataset without manipulated broods; exact *N*_females_ is unknown because this analysis includes broods whose mother could not be identified.

### CHANGE IN THE RELATIONSHIP BETWEEN OPTIMAL PHENOTYPE AND THE ENVIRONMENT

If the optimal laying date (LD_θ_, i.e., the laying date at which fitness is maximized) advanced at a faster rate over time than the observed laying date, this could lead to selection on (1) the reaction norm elevation, if the sensitivity of the optimal reaction norm to temperature (i.e., plasticity) remained constant over time, or (2) the slope, if the sensitivity of LD_θ_ to temperatures changed over time. We therefore determined LD_θ_ in each year and regressed it against the temperature cues used by the birds (i.e., the mean temperature across March 11–April 20). LD_θ_ was defined as the peak date of caterpillar biomass minus 33 (see Chevin et al. 2015). The rationale behind this was that it takes approximately 33 days from the laying date to the moment when chicks’ food requirements are highest, assuming a clutch size of 9–10, followed by 12–13 days of incubation, and a peak in food demands at chick age 9–11 d (e.g., Keller and Van Noordwijk 1994; Mols et al. 2005).

To test whether the optimal reaction norm changed over time, we divided the datasets into three equal‐interval time periods (spanning 14–15 years: 1973–1987, 1988–2001, 2002–2016; Table 1). We then fitted a linear model with LD_θ_ as a function of temperature and time period. We assessed statistical significance of model terms by bootstrapping (1000 iterations, bias‐corrected, and accelerated confidence intervals) estimates for each period, as well as the temperature slope. We then ran a model that contained an interaction between temperature and period and bootstrapped the change in the slope of the optimal reaction norm between periods to determine whether the slope changed over time. The year 1991 was excluded from this analysis as an exceptionally late frost spell in that year damaged all fresh oak leaves and hence the food peak was exceptionally late (see also Visser et al. 2002, 2006).

### SELECTION ON THE SLOPE AND ELEVATION OF THE GREAT TIT REACTION NORM

To estimate period‐specific (fecundity) selection on plasticity, we assigned each female with at least two breeding attempts to a breeding cohort (based on the first year a bird was observed breeding) and split the phenotypic dataset into three time periods as above. A female's lifetime reproductive success (LRS) was the sum of all recruits she produced over her lifetime. To avoid truncation of LRS of birds breeding in 1973, we added broods from earlier years (before 1973) to complete each individual's LRS. For the same reason, we removed all observations of birds from the 2013 breeding cohort or later, as some birds from these cohorts were still known to be breeding in 2017 and therefore had incomplete LRS. Hence, the periods in this analysis were 1969–1987, 1988–2001 and 2002–2012 (Table 1).

As pointed out above, some broods had been manipulated, likely affecting the female's fitness. Since we were interested in the lifetime fitness consequences of being more or less phenotypically plastic with respect to temperature, as well as having a higher or lower mean response (i.e., the elevation of the reaction norm), we opted to include manipulated broods in this analysis. The reason for this was that because the probability of having any of her broods manipulated increases with a female's age, discarding data from manipulated females would lead to a biased subset of shorter lived individuals. Consequently, the inclusion of manipulated broods in our analysis may create some noise in the selection estimates, but we nevertheless believe it is a superior approach to removing a nonrandom subset of individuals from the dataset.

For each of the three periods, we fitted a bivariate random regression model (RRM) in “MCMCglmm” (Hadfield 2010; Hadfield 2014) with laying date as a Gaussian and LRS as a (overdispersed) Poisson trait. Breaking down this bivariate model in scalar notation, the laying date (*z*) of the *i* ^th^ individual in the *j* ^th^ year in the *k* ^th^ nest box within the *l* ^th^ “environmental block” was modelled as

(1) $$\begin{array}{ccc} z_{\mathrm{ijkl}} & = & \alpha_{z}+a_{i}+b_{i}\left(T_{\mathrm{ij}}-{\bar{T}}_{i}\right)\\ & & +\;b{\bar{T}}_{i}+\mathrm{age}{}_{\mathrm{ij}}+nb_{k}+yr_{j}+e_{z,\mathrm{ijl}}\;, \end{array}$$

where $\alpha_{z}$ is the overall mean laying date (intercept), $a_{i}$ and $b_{i}$ are the individual intercept and slope, respectively, related to temperature (*T*) and *b* is the population‐level slope, $\mathrm{age}{}_{\mathrm{ij}}$ is the female's age (first‐year breeder, older, or unknown), $nb_{k}$ and $yr_{j}$ are the *k* ^th^ nest box and *j* ^h^ year, respectively (treated as random effects with estimated variance $nb_{k}∼N\left(0,\sigma_{nb}^{2}\right)$ and $yr_{j}∼N\left(0,\sigma_{yr}^{2}\right)$) and $e_{z,\mathrm{ijl}}$ is the residual term. This residual term was estimated as $e_{z,\mathrm{ijl}}∼N\left(0,\sigma_{e,l}^{2}\right)$, where *l* is one of two equal‐interval groups of years with similar temperatures, which was done to accommodate changes in residual variance along the temperature gradient (Lillehammer et al. 2009; Nicolaus et al. 2013). Temperature was divided into a within‐individual ($b_{i}\left(T_{\mathrm{ij}}-{\bar{T}}_{i}\right)$) and a between‐individual ($b{\bar{T}}_{i}$) component, following Van de Pol and Wright (2009). This method disentangles any effect of temperature on laying date caused by having observed certain individuals only under certain temperatures and others under different temperatures (a between‐subject effect of *T* on *z*) from a real, within‐subject effect (but note that for the purpose of estimating variance in intercepts, centring may not be desired in all context; see Kreft et al. (1995)).

A female's LRS (*W*) is described as

(2) $$\log\;\left(E{\left[W\right]}_{i}\right)=\alpha_{W}+c_{i}+e_{W,i},$$

where $\alpha_{W}$ is the overall mean LRS, $c_{i}$ is the individual intercept and $W_{i}∼\mathrm{Poisson}\left(E{\left[W\right]}_{i}\right)$. We set the residual term, $e_{W,i}$, to a fixed small variance (0.01) since LRS is a repeated measures trait and laying date is not; ideally, we would constrain variance to 0 (Brommer et al. 2012; Morrissey et al. 2012), but since the MCMC chain does not mix well under these conditions a minimum nonzero value needs to be assigned.

Variance in intercepts (*a*) and slopes (*b*) of the reaction norm (eq. (1)), as well as in intercepts of LRS (c;eq. (2)) were jointly fitted as the random effect “female identity,” such that individual values were estimated as

$${\left[\begin{array}{c} a\\ b\\ c \end{array}\right]}_{i}∼N\left(\left[\begin{array}{c} 0\\ 0\\ 0 \end{array}\right],{\left[\begin{array}{ccc} \sigma_{a}^{2} & \;\sigma_{a,b} & \;\sigma_{a,c}\\ \sigma_{a,b} & \;\sigma_{b}^{2} & \;\sigma_{b,c}\\ \sigma_{a,c} & \;\sigma_{b,c} & \;\sigma_{c}^{2} \end{array}\right]}_{i}\right),$$

that is, drawn from a 3 × 3 unstructured, phenotypic covariance matrix with the variance components on the diagonals and the covariances between them on the off‐diagonals.

To obtain independent samples from the MCMC sampling process, we used a thinning interval of 10^4^, a burn‐in period of 10^5^ and a total sample size of 10.1 × 10^6^ (i.e., an effective sample size of 1000). We used default normal priors for the fixed effects and parameter‐expanded priors for the random terms (V = diag(*n*), nu = *n*, alpha.mu = 0, alpha.V = diag(*n*)*25^2, *n* being the dimension of the matrix), following Hadfield (2014). For the residual variance in equation (1), we used inverse‐Wishart priors with V = diag(2) and nu = 0.002.

### PREDICTING CHANGES IN THE REACTION NORM

We used a multivariate version of the breeder's equation to predict changes in the reaction norm as a result of selection across the three time periods (Table 1). The evolutionary response to selection from one year to the next is defined as the trait heritability times the selection differential (*R* = *h* ^2^ *S*, i.e., the breeder's equation; Falconer and Mackay 1996). Even in its multivariate form (Lande 1979), however, this would require estimating the heritability of both components of the reaction norm (elevation and slope), which is not straightforward (Hadfield et al. 2010). We therefore used a derivation of the breeder's equation for reaction norms (Van Tienderen and Koelewijn 1994). In their Appendix 1, Van Tienderen and Koelewijn (1994) show that the selection gradients on reaction norm components, that is intercept and slope in our case, can be calculated from environment‐specific—here hence annual—selection gradients:

(3) $$\;\beta_{g}=\Phi^{t}\;\beta_{z},$$

where $\beta_{g}$ is the vector of selection gradients on intercept and slope, $\Phi^{t}$ is the transposed matrix of vectors consisting of the environmental values in environments *i…k* with a leading 1, and $\beta_{z}$ the vector of selection gradients in environments *i…k*. The (expected) genetic change in the breeding values for intercept and slope is given by the multivariate breeders’ equation:

(4) $$\Delta \;\bar{g}=G\beta_{g},$$

where $\Delta \bar{g}$ is the change in intercept and slope breeding values, and ***G*** the additive genetic variance‐covariance matrix for intercept and slope. Substituting equations (3) and (4) allows the change in reaction norm components ($\Delta \bar{g}$) as a result of selection in year *j* following

(5a) $$\Delta \;\bar{g}=G{\left[1,x_{j}\right]}^{t}\beta_{z_{j}},$$

where ${\left[1,x_{j}\right]}^{t}$ is a transposed vector characterising the environment in a given year ($x_{j}$), and $\beta_{z_{j}}$ the selection gradient. We then only need to accommodate generation time by multiplying the response by the proportion of the breeding population in environment *j*+1 represented by recruits ($p_{recr_{j+1}}$) and further halve the response because laying date is only expressed by females (following Gienapp et al. 2006):

(5b) $$\Delta \;\bar{g}=G{\left[1,x_{j}\right]}^{t}\beta_{z_{j}}\times p_{recr_{j+1}}\times \;0.5.$$

To estimate ***G*,** we fitted a univariate RRM on the entire dataset (i.e., all years and all observations) that included an additive genetic term (“animal model,” RRAM; Henderson 1988; Kruuk 2004) in “MCMCglmm”. Random effects were as in equation (1), with the addition of an additive genetic term (via the pedigree) to estimate additive genetic variance in laying date. The difference was that we allowed both the permanent environment term (i.e., female identity) and the additive genetic term to vary with grand‐mean‐centred, as opposed to individual‐mean‐centred, temperature. Thus, the RRAM took the form:

(6) $$\begin{array}{ccc} \;z_{\mathrm{ijkl}} & = & \alpha_{z}+a_{i}+A_{i}+\left(b+b_{i}+B_{i}\right)\left(T_{\mathrm{ij}}-\bar{T}\right)\\ & & +\;\mathrm{age}{}_{\mathrm{ij}}+nb_{k}+yr_{j}+e_{z,\mathrm{ijl}}, \end{array}$$

where definitions and indices are as for equation (1), but where the individual intercepts and slopes of the laying date–temperature reaction norm are now estimated on the grand‐mean‐centred temperature ($\bar{T}$) and partitioned into a permanent environment ($a_{i}$ and $b_{i}$) and additive genetic component ($A_{i}$ and $B_{i}$; breeding values). Phenotypic and additive genetic variance in these components was estimated using two separate, 2 × 2 unstructured phenotypic and additive genetic (***G***) variance–covariance matrices containing $\sigma_{a}^{2}$ and $\sigma_{b}^{2}$ or $\sigma_{A}^{2}$ and $\sigma_{B}^{2}$, respectively, as well as the covariance between each component. The residual term was estimated from a 4 × 4 matrix to allow for independent and heterogeneous variance as in equation (1), where 4 is the rounded number of years divided by 10, grouped based on temperature. We extracted posterior medians from ***G***, along with 95% HPDIs (note that because variance estimates are constrained to be positive, they have a skewed posterior sample distribution when close to 0, hence making the median a more appropriate point summary than the mean). We applied the same prior structure and sampling procedures as for equation (1). Estimates resulting from the RRAM were robust to excluding individuals with only one or only two breeding records.

To estimate the selection gradient, $\beta_{z_{j}}$, we ran annual Generalized Additive Models (GAMs; package “mgcv”; Wood 2017) on unmanipulated broods, with annual reproductive success (ARS; number of recruited offspring) as response variable with a negative binomial distribution and laying date as the predictor variable. We then used the “gam.gradients” function from the “gsg” package (Morrissey and Sakrejda 2015) to calculate $\beta_{z_{j}}$ (see Lande and Arnold 1983) along with the standard error through parametric bootstrapping (1000 iterations). Females with unknown identity were included in these analyses as they comprise a potentially biased subset of individuals that laid their eggs too early or too late, in some cases leading to brood desertion before the nestling stage when adults could be identified.

With ***G*** and $\beta_{z}$ in place, we could predict the evolution of the reaction norm throughout the study period. Since the reaction norm has to be estimated across years (because each year has only one temperature average and one breeding event), we could not predict change from the first year onward. Instead, we estimated the observed population reaction norm across years in period 1 (see Table 1) using a slightly modified version of equation (1) ( $z_{\mathrm{ijkl}}=\alpha_{z}+a_{i}+b\left(T_{\mathrm{ij}}-{\bar{T}}_{i}\right)+b{\bar{T}}_{i}+\mathrm{age}{}_{\mathrm{ij}}+nb_{k}+yr_{j}+e_{z,\mathrm{ijl}}$, i.e. a random‐intercept model) and predicted the evolutionary outcome of selection in all subsequent years (i.e., excluding years in period 1) using equation (5b). Note that the predicted reaction norm after selection is then a property of a single year and will hence never accurately match the observed reaction norm, which is necessarily estimated across multiple years. We therefore view the predicted change as the upper limit of possible change within the studied period.

Since both ***G*** and ***β***_***z***_ came with estimation errors, we accommodated uncertainty in predicted cumulative change in the following way. We used the upper and lower 95% HPDI of ***G*** to calculate the variance (*V*) for $G{\left[1,x_{j}\right]}^{t}$ in each year by squaring its standard error (half the 95% HPD range divided by 1.96); similarly, we squared the standard error of $\beta_{z_{j}}$ to get its variance. The standard error of the full product was then calculated following simple error‐propagation rules (Taylor 1997):

(7) $$\mathrm{SE}{}_{\Delta \bar{g}}=\sqrt{{\left(G{\left[1,x_{j}\right]}^{t}\right)}^{2}V_{\beta_{z_{j}}}+{\beta_{z_{j}}}^{2}V_{G{\left[1,x_{j}\right]}^{t}}}\;,$$

which, in the case of equation (5b), was in turn multiplied by $p_{recr_{j+1}}\times \;0.5$. We calculated the cumulative difference across years ($\Delta {\bar{g}}_{cum.}$) by summing $\Delta \bar{g}$ of each year; the 95% CI was calculated as $\Delta {\bar{g}}_{cum.}\pm$ $1.96\;\times {\left(\mathrm{SE}{{}_{\Delta {\bar{g}}_{1}}}^{2}+\cdots +\mathrm{SE}{{}_{\Delta {\bar{g}}_{n}}}^{2}\right)}^{0.5}$, where *n* is the number of years in period 2 and 3 combined. We need to make the cautionary note that although this way of calculating of errors should work satisfactorily when posterior distributions approximate normality, it may cause upward bias (i.e., be conservative) for variance estimates that are skewed because they are bounded at 0.

We visually compared the predicted reaction norm with the observed reaction norms in each of the three periods, which we estimated in “MCMCglmm” using a trimmed version of equation (6) as described above and using the same priors and MCMC sampling procedures as described for equation (1). Although we cannot compare the predicted and observed reaction norms quantitatively for the reasons given above, we do present them alongside one another as the latter serves as an empirical validation of the former.

## Results

### CHANGE IN OPTIMAL REACTION NORM OVER TIME

**Figure 1:**
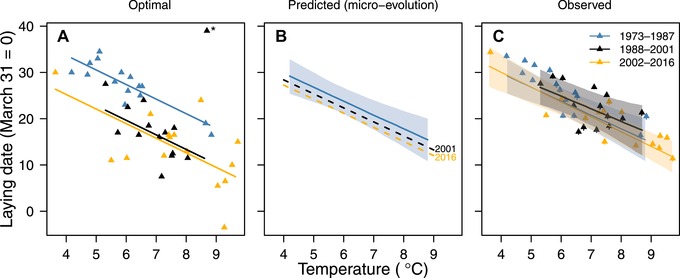
Optimal (A), predicted (B), and observed (C) reaction norms of laying date against spring temperature in three consecutive periods (blue: 1973–1987; black: 1988–2001; orange: 2002–2016) in the HV great tit population. (A) The optimal laying date for each year was the caterpillar peak date minus 33 days. Lines are estimates from linear regressions, excluding 1991 (marked by an asterisk) because late frost damaged the oak leaves in that year. (B) The black and orange dashed lines are the predicted evolutionary deviation from the observed reaction norm in period 1 (solid blue line) by the end of the second (2001) and third (2016) time period, respectively, based on cumulative change due to annual selection (eq. (5b); see Methods for interpretation). (C) Observed laying dates are yearly averages (±1 SEM); solid lines and shadings are the regression lines and 95% HPDI regions from a univariate mixed‐effects model of laying date against temperature (i.e., the mean individual‐level slope).

We found that the optimal reaction norm for laying date (estimated as the date of the caterpillar biomass peak versus the temperatures in the time window as used by great tits to time their reproduction) changed to earlier dates over three distinct periods (Fig. 1A); the optimal phenotype (LD_θ_) advanced by 7.72 days (95% CI: [–10.89, –4.79]) from the first to the second period, and by 8.45 days (95% CI: [–12.75, –5.13]) from the first to the third period. There was, however, no interaction between temperature and time period (change in slope coefficient from first to second period: –1.54 [–4.19, 2.15]; from the first to the third period: 0.58 [–2.10, 3.31]), indicating that the response of LD_θ_ to temperature did not change over the three periods (slope coefficient across all years: –3.15 [–4.36, –1.76] days/°C). Besides the mean, interannual variance in optimal laying date changed across the three periods (24.31 [10.82, 50.36], 29.98 [15.09, 64.27] and 63.70 [30.56, 149.37], respectively).

### SELECTION ON THE GREAT TIT REACTION NORM

Along with a change to earlier dates in the elevation of the optimal reaction norm, we found statistical evidence for directional selection on the elevation of the great tit reaction norm in period 2; that is, lifetime reproductive success (LRS) covaried negatively with the elevation, indicating selection for a lower reaction norm (Table 2). Despite overwhelmingly negative annual selection gradients (Table S1), this selection on the intercept weakened in period 3, potentially related to the high variability in phenotypic optima in that period. We found no evidence for directional selection on slopes, and estimates of slope variation were overall small and zero‐bound (Table 2). This low slope variance was also found when we used the entire 1973–2016 dataset (Table 3; see Discussion).

**Table 2:** Posterior medians (and 95% HPDIs) of the phenotypic (co)variance matrix resulting from an analysis of selection on the reaction norm of laying date (LD) against temperature (via lifetime reproductive success (LRS); eqs. (1) and (2)) for three distinct cohort periods in the HV great tit population, excluding females with only one observation (see Table 1 for sample sizes)

	LD_intercept_	LD_slope_	LRS
*Period 1*			
LD_intercept_	6.48 (4.78, 8.09)		
LD_slope_	0.29 (–0.26, 1.11)	0.15 (0.00, 0.77)	
LRS	–0.32 (–0.74, 0.11)	–0.02 (–0.25, 0.20)	1.12 (0.89, 1.40)
*Period 2*			
LD_intercept_	9.88 (7.67, 12.62)		
LD_slope_	1.00 (–0.32, 2.80)	0.43 (0.00, 1.61)	
LRS	**–0.98 (–1.58, –0.32)**	–0.24 (–0.76, 0.15)	1.51 (1.14, 1.97)
*Period 3*			
LD_intercept_	7.92 (5.52, 10.70)		
LD_slope_	–0.24 (–1.04, 0.55)	0.21 (0.00, 0.69)	
LRS	–0.53 (–1.15, 0.13)	–0.22 (–0.46, 0.05)	1.23 (0.85, 1.62)

Variance estimates are given on the diagonals, whereas covariance estimates are on the off‐diagonals. Covariance estimates whose HPDI did not include zero are marked in bold.

**Table 3:** Model estimates resulting from the RRAM (eq. (6)) quantifying the ***G*** matrix for great tit laying date in the HV population between 1973 and 2016 (see Table 1 for sample sizes)

Parameter	Posterior median	95% HPDI	
*Fixed effects*			
Intercept	21.84	20.82	22.77
Age (old)	—	—	—
Age (unkn.)	1.27	0.42	2.13
Age (young)	1.78	1.51	2.05
Temperature (*T_c_*)	–3.28	–3.92	–2.68
*Random effects*			
$V_{year}$	9.65	5.92	14.45
***V***_***PE***_	**3.24**	**0.88**	**5.68**
$\mathrm{Cov}(\beta 0_{\mathrm{PE}},\beta 1_{T_{c}})$	**0.06**	**–0.16**	**0.47**
$V_{\mathrm{PE}\times T_{c}}$	**0.02**	**0.00**	**0.15**
***V***_***A***_	**4.38**	**1.98**	**6.70**
$\mathrm{Cov}(\beta 0_{A},\beta 1_{T_{c}})$	**0.07**	**–0.20**	**0.43**
$V_{A\times T_{c}}$	**0.02**	**0.00**	**0.15**
$V_{\mathrm{NB}}$	1.22	0.85	1.66
$V_{R_{3.64-5.16C}}$	11.09	9.40	13.05
$V_{R_{5.16-6.68C}}$	13.63	12.20	14.99
$V_{R_{6.68-8.20C}}$	19.21	17.35	21.17
$V_{R_{8.20-9.72C}}$	19.27	16.32	21.90

$T_{c}$ = mean‐centred temperature; $V_{x}$ = variance component associated with each random effect (*PE* = permanent environment, *A* = additive genetic, *NB* = nest box; *R* = residual); Cov(β0,β1) = intercept–slope covariance.

The permanent‐environment and additive genetic (co)variance components are marked in bold.

### PREDICTED AND OBSERVED GREAT TIT REACTION NORMS

There was ample additive genetic variation in the elevation, but not the slope, of the great tit reaction norm (Table 3). Based on selection patterns in Table 2, we may expect the reaction norm to evolve toward an earlier average over time, but its slope to change only marginally because of the weak selection on it. Using the quantitative genetic model (eq. (5b)), we found that both the elevation and the slope of the reaction norm were expected to evolve only marginally over time (Fig. 1B; cumulative change in elevation across years in period 2 and 3: –2.34 [–4.20, –0.48] days; in slope: –0.04 [–0.09, 0.02]; see Table S1 for annual predicted responses). The small predicted response in the elevation—compared to the change in the optimal reaction norm—despite strong selection in period 2 was due to the generation time of about two years and the sex limitation of the trait; when we disregarded these factors (eq. (5a)), the predicted response in elevation was substantially stronger (cumulative change in elevation: –10.30 [–18.63, –1.98] days; in slope: –0.16 [–0.40, 0.08]; Table S1).

In close agreement with the predicted reaction norms, the observed reaction norm showed no distinct advancement over the three periods (Fig. 1C), with largely overlapping 95% HPDIs for the intercepts (posterior medians and 95% HPDIs for period 1–3: 41.80 [33.40, 49.29], 41.27 [30.72, 53.50], and 42.73 [35.85, 50.81]).

## Discussion

We studied whether the optimal reaction norm of great tit laying date against temperature changed over three consecutive time periods in a long‐term study population and whether this resulted in intensified directional selection on—and an evolutionary response in—its two components (elevation and slope, i.e., the sensitivity to the environmental variable). We found that, whereas the optimal laying date (as predicted by spring temperatures and determined by the timing of the caterpillar peak) across the temperature range (the elevation of the optimal reaction norm) advanced over the past decades, the sensitivity of the optimal laying date to temperature (the slope of the optimal reaction norm) did not change. In agreement with this, there was selection on the great tit reaction norm elevation, but not on the slope, in the second third of our time series, but this selection waned in the third period, potentially due to the increased variability in phenotypic optima (Fig. 1A). Despite directional selection, we predicted quantitatively that the elevation of the reaction norm would shift only marginally over time (a maximum of a few days), simply because of the low heritability, the generation time of about two years (Garant et al. 2004; Kvist et al. 2007) and the sex limitation of laying date (Caro et al. 2009). Indeed, the actual (observed) reaction norm did not change over time, neither in slope nor elevation. Our results suggest that, despite the apparent lack of an evolutionary response in our population, adaptation of timing of breeding to climate change can only occur through evolution of the elevation of the reaction norm, as evolution of increased (or decreased) phenotypic plasticity is not possible.

Selection on plasticity in our study population was limited because the slope of the optimal reaction norm did not change over time. However, even if there had been substantial directional selection on the slope, the response to selection would have been weak as there was very little variation in individual reaction norm slopes, both phenotypically and additive genetically (Tables 2 and 3). This qualitative statement can be quantified by comparing the variances in environment‐specific expected values of laying date attributable separately to variation in reaction norm elevations (i.e., the estimates from Table 3) and slopes (i.e., estimated variance multiplied by the variance in centred temperatures; see Appendix 7 in Morrissey and Liefting 2016). This was 3.24 [0.88, 5.68] and 4.38 [1.98, 6.70] for the phenotypic and additive genetic intercepts, respectively, whereas it was only 0.05 [0.00, 0.34] and 0.04 [0.00, 0.34] for the respective slopes. Thus, the contribution of variance in slopes to variance in expected laying dates in a given environment was negligible.

The lack of variation in (phenotypic) plasticity slopes (I × E) contradicts previous work on our study population (Nussey et al. 2005b; Husby et al. 2010, 2011). The source of this discrepancy lies in the residual variance structure of the random regression models. Besides the problems inherent to using BLUPs in this context, Nussey et al. (2005b) fitted homogeneous residual variance, thereby forcing any heterogeneity in residual variance caused by the environment (i.e., temperature) to be estimated by the phenotypic covariance matrix and hence inflating estimates of I × E. Husby et al. (2010, 2011), on the other hand, fitted a heterogeneous residual structure but grouped years by decade, assuming that variance in laying date increased over the years because temperature increased over time. This assumption does not hold up, however, as we found that the variance in laying date in HV correlated very weakly with year (slope = 0.001 [–0.22, 0.22]; *r* ^2^ = 0.00 [0.00, 0.001]), whereas it correlated reasonably well with temperature (2.40 [0.70, 4.49]; *r* ^2^ = 0.13 [0.01, 0.36]). In general, it would make more sense to partition residual variance based on years with similar environments rather than based on decades. Indeed, when we fitted the model of equation (6) (but dropping the G × E term for efficiency) on the same data subset (1973–2006) as in Husby et al. (2010, 2011) and partitioned residual variance into three groups by decade, we found substantial individual variation in slopes (posterior median slope variance and 95% HPDI: 0.91 [0.36, 1.53]). When we refitted the model on the same data subset (1973–2006) with residual variance partitioned into three groups based on temperature, however, the slope variance decreased substantially (0.25 [0.00, 0.87]). These results were confirmed by simulations; when we simulated a population with a small slope variance, specifying the incorrect residual structure in the random regression model led to upward bias in the variance estimate and a high false‐positive rate (Supplementary Info 2, Fig. S1). Thus, we conclude that variation in plasticity in HV really is limited, reinforcing the notion that, despite previous studies (Nussey et al. 2005b), there can be only limited response to selection on plasticity in this population (cf. e.g., Brommer et al. 2012; Hayward et al. 2014).

Although we can only speculate about the reasons for an apparent lack of I × E and G × E in our population, previous simulation studies suggest it is unlikely that we had limited statistical power (or precision in our case) to detect either (Martin et al. 2011; Van de Pol 2012). Illustrative in this respect are the narrow 95% HPDIs for slope variances (Table 3), which seem to indicate that the RRAM was able to estimate I × E (strictly speaking PE × E and G × E) with a fair amount of precision. To put this in perspective, when we applied a version of the model of equation (6) (that included individual‐mean‐centred temperatures) to another of our long‐term great tit study populations on the Dutch island of Vlieland (VL), we found substantial evidence for I × E in laying date (posterior median slope variance and 95% HPDI: 1.32 [0.53, 1.89]), but not G × E (0.10 [0.00, 0.67]; unpublished results), both estimated with substantially lower precision (the zero constraint of the estimates notwithstanding). The HV and VL datasets are quite similar in terms of the number of individuals and observations and we should therefore have been able to detect I × E in HV if it was really there. Since the pedigree in VL is more informative (“deeper”) than the one in HV because of limited immigration from the mainland (Gienapp et al. 2013b; Postma and Van Noordwijk 2005), the VL data may be better suited to test for G × E, but even there G × E appeared to be limited. (No caterpillar biomass data are available for that population, making it an unsuitable dataset for the purpose of this article.) This apparent absence of G × E is consistent with the notion that G × E in general is hard to detect in wild populations when not in an experimental setting (Gienapp and Brommer 2014; Wood and Brodie III 2016; Hayward et al. 2018; Ramakers et al. 2018). Nevertheless, since I × E is the upper limit of G × E (Gienapp and Brommer 2014), the absence of I × E in our study system in HV suggests that G × E is absent. The reason for this absence may be that early‐spring temperatures have historically been highly predictive of the food peak and selection for being well matched with this peak has been strong (Reed et al. 2013b; Visser et al. 2006), possibly eroding (genetic) variation in plasticity in laying date over time (cf. Tomkins et al. 2004; Turelli and Barton 2004).

Despite selection on, and ample additive genetic variation in, the elevation of the reaction norm (Tables 1 and S1), the observed, period‐specific reaction norms changed only marginally over the course of time, as was also predicted from the quantitative genetic model (eq. (5b)). A close look at Figure 1C reveals that the population is “sliding” up and down the same reaction norm; that is, the observed temperature range has become wider and individuals are still using these temperatures to time the onset of reproduction (see also Visser et al. 2006). Clearly, given the increase in mismatch with the caterpillar peak in this population, this is not an adaptive strategy (Reed et al. 2013b; Thomas et al. 2001); the population needs to evolve a lower elevation to become better matched with the caterpillar peak. The apparent lack of such a shift is most likely explained by the low heritability of elevation, the generation time and the sex limitation of laying date, as shown by our quantitative genetic model (eq. (5b)). Additionally, increased variability in phenotypic optima in the last third of the study period may further hamper adaptation as early‐breeding genotypes no longer consistently have a reproductive advantage.

In the quantitative genetic model, we used annual phenotypic selection gradients to predict the change in the reaction norm from one year to the next. One concern with using phenotypic (annual) selection gradients is that estimates of selection in reality reflect an environmental covariance between the trait and fitness or selection on a correlated trait (Lande and Arnold 1983; Price et al. 1988; Hadfield 2008). In our population, however, we know that such environmental bias in selection is limited and that estimates of selection at the phenotypic and genetic level are very similar (Gienapp et al. 2006; Reed et al. 2016). Thus, the necessary ingredients for genetic adaptation—genetic variation and selection—are real and present in HV, but evolutionary change is simply too small to be detected due to the large environmental variation in laying date among years (see also discussion in Gienapp et al. 2006). Such small rates of adaptation can put a strain on population persistence in the longer run as adaptation continues to be outpaced by climate change (Gienapp et al. 2013a; Carlson et al. 2014; Visser and Gienapp, unpubl. ms.; but see Reed et al. 2013a,b).

Many populations have thus far responded to the increased mismatch with the phenology of their food through phenotypic plasticity (Charmantier and Gienapp 2014; Merilä and Hendry 2014), but it has been predicted that this cannot be sufficient in the long run as climate change continues to disrupt synchrony between trophic levels (Visser et al. 2004; Visser 2008; Thackeray et al. 2010; Carlson et al. 2014; Thackeray et al. 2016; Visser and Gienapp, unpubl. ms.). Consequently, we need to know whether an absence of G × E in phenology as reported here is the general case or an exception. As pointed out above, such studies in natural populations are rare (but see Ramakers et al. 2018), which is likely due to the logistical challenges of obtaining the necessary pedigree, so far constraining these studies to mainly birds and mammals. Replacing the observational, social pedigree by relatedness estimated from genetic markers has earlier been suggested (Moore and Kukuk 2002; Ritland 1996), but applications of this approach remained unsuccessful (Coltman 2005; Garant and Kruuk 2005; Csilléry et al. 2006). However, with the advent of high‐throughput, high‐density genotyping to “nonmodel” species, “genomic” relatedness estimates should have become sufficiently reliable to replace pedigrees (Gienapp et al. 2017) and thereby allow us to study genetic variation in and selection on phenotypic plasticity both in labile and fixed traits in a broader range of taxa.

To date, evidence for successful evolutionary rescue through a genetic shift in the reaction norm remains rare (Merilä and Hendry 2014). One textbook example of successful evolutionary rescue is that of the great tit's most important food source, caterpillars of the winter moth (*O. brumata*); three Dutch populations have now restored the match of their hatching date with oak (*Quercus robur*) bud burst through a genetic shift in the elevation of the reaction norm (Van Asch et al. 2013). It remains unclear whether such a change will be also observable in vertebrate populations, as the methods thus far deployed have been largely insufficient to infer evolutionary change (Merilä and Hendry 2014). We provide an important step to this discussion by using rigorous statistical tools to reveal the evolutionary potential of a key life‐history trait in a reaction norm context.

Associate Editor: M. Morrissey

Handling Editor: P. Tiffin

## Supporting information


**Table S1**. Annual selection gradients (β*_Z_*), absolute (T) and mean‐centred (T_c_) mean spring temperature, and the predicted annual evolutionary changes (Δ) in the reaction norm elevation (intercept, Int) and slope (Slp), along with standard errors (SE), as a result of annual selection on great tit laying date from the second time period onward (i.e. excluding 1973–1987 because the ‘initial’ reaction norm was estimated over these first 15 years).
**Figure S1**. Results of the simulation testing the effect of the residual structure of the random regression model on the estimate of variance in slopes (a, c, e) and the respective statistical power to detect this variance (b, d, f).

Supporting Information
