# Supplementary material for: Phenological mismatch drives selection on elevation, but not on slope, of breeding time plasticity in a wild songbird
Source: Evolution. 2018 Dec 21;73(2):175–87. doi: 10.1111/evo.13660 (PMC6519030; doi:10.1111/evo.13660)
Supplement: Supplementary file 1 — Table S1. Annual selection gradients (βZ), absolute (T) and mean‐centred (Tc) mean spring temperature, and the predicted annual evolutionary changes (Δ) in the reaction norm elevation (intercept, Int) and slope (Slp), along with standard errors (SE), as a result of annual selection on great tit laying date from the second time period onward (i.e. excluding 1973–1987 because the ‘initial’ reaction norm was estimated over these first 15 years). Figure S1. Results of the simulation testing the effect of the residual structure of the random regression model on the estimate of variance in slopes (a, c, e) and the respective statistical power to detect this variance (b, d, f). [file EVO-73-175-s001.docx]

Supplementary Information

“Phenological mismatch drives selection on elevation, but not on slope, of breeding time plasticity in a wild songbird”

**S1 – Predicting evolution of the reaction norm**

**Table S1.** Annual selection gradients ($\beta_{z}$), absolute (T) and mean-centred (T_c_) mean spring temperature, and the predicted annual evolutionary changes ($\Delta$) in the reaction norm elevation (intercept, Int) and slope (Slp), along with standard errors (SE), as a result of annual selection on great tit laying date from the second time period onward (i.e. excluding 1973–1987 because the ‘initial’ reaction norm was estimated over these first 15 years). Predicted changes were estimated while accounting for generation time and the sex limitation of laying date (**a**) and when not accounting for these factors (**b**). See main text and eqn. 5a and 5b for details.

| Year | $\beta_{z}$ | $\mathrm{SE}_{\beta_{z}}$ | T (°C) | T_c_ | (a) Generation time and sex limitation included | | | |  | (b) Generation time and sex limitation not included | | | |
| --- | --- | --- | --- | --- | --- | --- | --- | --- | --- | --- | --- | --- | --- |
|  |  |  |  |  | ΔInt | SE_Int_ | ΔSlp | SE_Slp_ |  | ΔInt | SE_Int_ | ΔSlp | SE_Slp_ |
| 1973 | 0.007 | 0.049 | 5.05 | –1.84 | - | - | - | - |  | - | - | - | - |
| 1974 | –0.010 | 0.028 | 8.65 | 1.76 | - | - | - | - |  | - | - | - | - |
| 1975 | 0.025 | 0.036 | 4.18 | –2.71 | - | - | - | - |  | - | - | - | - |
| 1976 | –0.018 | 0.021 | 5.96 | –0.93 | - | - | - | - |  | - | - | - | - |
| 1977 | –0.045 | 0.035 | 5.11 | –1.78 | - | - | - | - |  | - | - | - | - |
| 1978 | 0.010 | 0.048 | 5.62 | –1.27 | - | - | - | - |  | - | - | - | - |
| 1979 | –0.006 | 0.022 | 5.84 | –1.05 | - | - | - | - |  | - | - | - | - |
| 1980 | –0.067 | 0.041 | 6.06 | –0.83 | - | - | - | - |  | - | - | - | - |
| 1981 | –0.028 | 0.040 | 8.84 | 1.95 | - | - | - | - |  | - | - | - | - |
| 1982 | –0.074 | 0.034 | 6.43 | –0.46 | - | - | - | - |  | - | - | - | - |
| 1983 | 0.000 | 0.073 | 6.47 | –0.42 | - | - | - | - |  | - | - | - | - |
| 1984 | 0.037 | 0.744 | 4.71 | –2.18 | - | - | - | - |  | - | - | - | - |
| 1985 | –0.204 | 0.084 | 6.53 | –0.36 | - | - | - | - |  | - | - | - | - |
| 1986 | –0.036 | 0.109 | 4.79 | –2.10 | - | - | - | - |  | - | - | - | - |
| 1987 | –0.165 | 0.060 | 6.14 | –0.75 | - | - | - | - |  | - | - | - | - |
| 1988 | –0.191 | 0.132 | 7.10 | 0.21 | –0.070 | 0.130 | –0.001 | 0.003 |  | –0.792 | 1.470 | –0.013 | 0.038 |
| 1989 | –0.032 | 0.043 | 7.30 | 0.41 | –0.024 | 0.030 | 0.000 | 0.001 |  | –0.153 | 0.192 | –0.003 | 0.007 |
| 1990 | –0.079 | 0.031 | 7.60 | 0.71 | –0.068 | 0.033 | –0.001 | 0.003 |  | –0.358 | 0.176 | –0.006 | 0.016 |
| 1991 | 0.006 | 0.043 | 8.69 | 1.80 | 0.007 | 0.023 | 0.000 | 0.001 |  | 0.056 | 0.174 | 0.001 | 0.005 |
| 1992 | –0.059 | 0.074 | 6.58 | –0.31 | –0.041 | 0.200 | –0.001 | 0.003 |  | –0.205 | 0.992 | –0.003 | 0.015 |
| 1993 | –0.165 | 0.081 | 7.52 | 0.63 | –0.131 | 0.078 | –0.002 | 0.006 |  | –0.693 | 0.413 | –0.012 | 0.030 |
| 1994 | –0.200 | 0.349 | 6.42 | –0.47 | –0.228 | 0.551 | –0.003 | 0.010 |  | –0.878 | 2.122 | –0.011 | 0.040 |
| 1995 | –0.078 | 0.073 | 6.05 | –0.84 | –0.048 | 0.062 | –0.001 | 0.002 |  | –0.239 | 0.309 | –0.003 | 0.008 |
| 1996 | –0.118 | 0.089 | 5.31 | –1.58 | –0.146 | 0.121 | –0.001 | 0.004 |  | –0.524 | 0.435 | –0.004 | 0.013 |
| 1997 | –0.075 | 0.088 | 6.75 | –0.14 | –0.086 | 0.277 | –0.001 | 0.005 |  | –0.309 | 0.994 | –0.004 | 0.018 |
| 1998 | –0.188 | 0.075 | 7.55 | 0.66 | –0.203 | 0.274 | –0.004 | 0.010 |  | –0.827 | 1.119 | –0.015 | 0.040 |
| 1999 | –0.052 | 0.087 | 8.05 | 1.16 | –0.050 | 0.550 | –0.001 | 0.011 |  | –0.235 | 2.578 | –0.005 | 0.052 |
| 2000 | –0.092 | 0.026 | 7.18 | 0.29 | –0.112 | 0.048 | –0.002 | 0.004 |  | –0.364 | 0.156 | –0.006 | 0.014 |
| 2001 | –0.114 | 0.037 | 5.72 | –1.17 | –0.096 | 0.038 | –0.001 | 0.003 |  | –0.466 | 0.185 | –0.005 | 0.013 |
| 2002 | –0.008 | 0.027 | 7.42 | 0.53 | –0.004 | 0.034 | 0.000 | 0.001 |  | –0.013 | 0.110 | 0.000 | 0.002 |
| 2003 | –0.151 | 0.063 | 7.26 | 0.37 | –0.178 | 0.088 | –0.003 | 0.007 |  | –0.698 | 0.347 | –0.012 | 0.028 |
| 2004 | –0.023 | 0.030 | 8.02 | 1.13 | –0.025 | 0.042 | 0.000 | 0.001 |  | –0.078 | 0.133 | –0.002 | 0.004 |
| 2005 | –0.055 | 0.047 | 9.53 | 2.64 | –0.059 | 0.063 | –0.002 | 0.004 |  | –0.223 | 0.240 | –0.006 | 0.014 |
| 2006 | –0.111 | 0.060 | 6.02 | –0.87 | –0.125 | 0.070 | –0.001 | 0.004 |  | –0.470 | 0.265 | –0.005 | 0.014 |
| 2007 | –0.205 | 0.069 | 9.27 | 2.38 | –0.206 | 0.101 | –0.005 | 0.012 |  | –0.918 | 0.450 | –0.023 | 0.052 |
| 2008 | –0.119 | 0.050 | 5.50 | –1.39 | –0.127 | 0.067 | –0.001 | 0.003 |  | –0.461 | 0.243 | –0.004 | 0.012 |
| 2009 | –0.044 | 0.052 | 9.30 | 2.41 | –0.050 | 0.059 | –0.001 | 0.003 |  | –0.195 | 0.231 | –0.005 | 0.012 |
| 2010 | –0.003 | 0.039 | 8.49 | 1.60 | –0.001 | 0.050 | 0.000 | 0.001 |  | –0.002 | 0.181 | 0.000 | 0.004 |
| 2011 | –0.082 | 0.032 | 9.05 | 2.16 | –0.092 | 0.050 | –0.002 | 0.005 |  | –0.325 | 0.177 | –0.008 | 0.018 |
| 2012 | 0.011 | 0.045 | 7.45 | 0.56 | 0.012 | 0.039 | 0.000 | 0.001 |  | 0.062 | 0.203 | 0.001 | 0.004 |
| 2013 | –0.095 | 0.060 | 3.64 | –3.25 | –0.078 | 0.052 | 0.000 | 0.001 |  | –0.392 | 0.260 | 0.000 | 0.003 |
| 2014 | –0.092 | 0.046 | 9.72 | 2.83 | –0.067 | 0.047 | –0.002 | 0.004 |  | –0.359 | 0.253 | –0.010 | 0.022 |
| 2015 | –0.004 | 0.045 | 6.81 | –0.08 | 0.007 | 0.037 | 0.000 | 0.001 |  | 0.038 | 0.190 | 0.001 | 0.003 |
| 2016 | –0.057 | 0.032 | 7.60 | 0.71 | –0.053 | 0.031 | –0.001 | 0.002 |  | –0.281 | 0.164 | –0.005 | 0.012 |
| **Total** | **-** | **-** | **-** |  | –2.341 | 0.948 | –0.037 | 0.027 |  | –10.304 | 4.248 | –0.164 | 0.122 |

**S2 – Simulation: testing the effect of the residual variance structure on detecting I×E**

The lack of between–individual variation in reaction slopes (I×E) in the Hoge Veluwe (HV) population contradicts previous studies (Nussey *et al.* 2005; Husby *et al.* 2010; Husby, Visser & Kruuk 2011). Here, we show by simulation that specifying the residual variance structure of the random regression model incorrectly will lead to wrong inferences about the presence of I×E (for simplicity, we disregard G×E here, but the same reasoning applies).

*The model*

We partly used the posterior estimates from the random regression animal model presented in Table 3 (main text) as basis for the simulation. We simulated a population of 1000 individuals with each roughly 2.6 observations (which is the mean no. observations per individual in HV when disregarding females that bred only once), distributed over 44 environments (years). We tested the effect of different residual structure on three scenarios, i.e. with small (0.1), intermediate (0.5) and large (1) variation in reaction norm slopes.

First, we drew 44 random temperatures from a normal distribution based on real temperature data (mean = 6.9, sd = 1.5). We then randomly drew a number of observations for each individual from a Poisson distribution, such that the mean per individual approximated 2.6. We then randomly assigned each individual to a cohort (and hence the temperatures they were exposed to). We randomly assigned a reaction norm to each individual by drawing an intercept ($a_{i}$) and a linear slope ($b_{i}$) from a random, normal distribution with mean = 0 and $\sigma^{2}$ = 3.5 and either 0.1, 0.5 or 1, respectively. Temperature values were individually mean–centred ($T_{i}$), and phenotypes (laying date or LD) were derived as ${LD}_{ij}=a_{i}+b_{i}T_{ij}+{bT}_{av,i}+e_{ij}$, where $T_{av,i}$ is the average temperature experienced by the individual and $e_{ij}$ is the error term. The error term was randomly drawn from a normal distribution with mean = 0 and $\sigma^{2}$ = 10.8, 13.4, 19.2 or 19.9, depending on the temperature in that environment; these values were taken from Table 3 (main text) and were used to make variance in LD dependent on temperature, as is the case in our great tit population.

For each slope variance scenario (small, intermediate, or large), we fitted three mixed–effects models in ASReml–R (Butler *et al.* 2009; Gilmour *et al.* 2009), each a variation on $LD$ *~ individual–centred temperature + individual–mean temperature, random = Individual×individual–centred temperature* (i.e. 9 scenarios): (i) with residual variance partitioned into four temperature blocks, i.e. years equally divided based on temperature; (ii) residual variance partitioned into four ‘decadal’ blocks, i.e. based on consecutive years; and (iii) homogeneous residual structure. Each model was tested against a simpler model ($LD$ *~ individual–centred temperature + individual–mean temperature, random = Individual*) to test for significance of I×E using likelihood–ratio tests with 1 degree of freedom. Starting values in ASReml–R were set such that they matched the input values.

The whole procedure was iterated 1000 times. The R script has been uploaded as a separate text file.

*Results and discussion*

As expected, slope variance estimates matched the input values nicely when we used heterogeneous residual variance based on temperature blocks in each of the three slope variance input scenarios (Figure S1a, c and e). When slope variance was small (Fig S1a), specifying the wrong residual structure inflated the estimates; with larger input values, however, this bias largely disappeared (panels c and e). Again as expected, power to detect I×E at a low slope variance was limited when using the appropriate residual structure, but strikingly, specifying the wrong residual structure led to large false positive rates (Fig S1b). Again, this discrepancy between models disappeared as true slope variance increased (panels d and f).

We conclude that specifying the right residual structure is essential for making correct inference of the presence of I×E (or G×E). This in itself is not a new insight (e.g. Gienapp & Brommer 2014), but it stresses the importance of carefully assessing which parameter drives variation in a phenotype. In the great tit example, this is clearly temperature and not year as a proxy for temperature. When true variance in slopes is small, therefore, an incorrectly specified residual structure will lead to both quantitatively and qualitatively different results (i.e. whether or not there is I×E). When variance is substantial, however, the chance of making a qualitatively (and perhaps quantitatively) wrong inference may in fact be reasonably small.

**
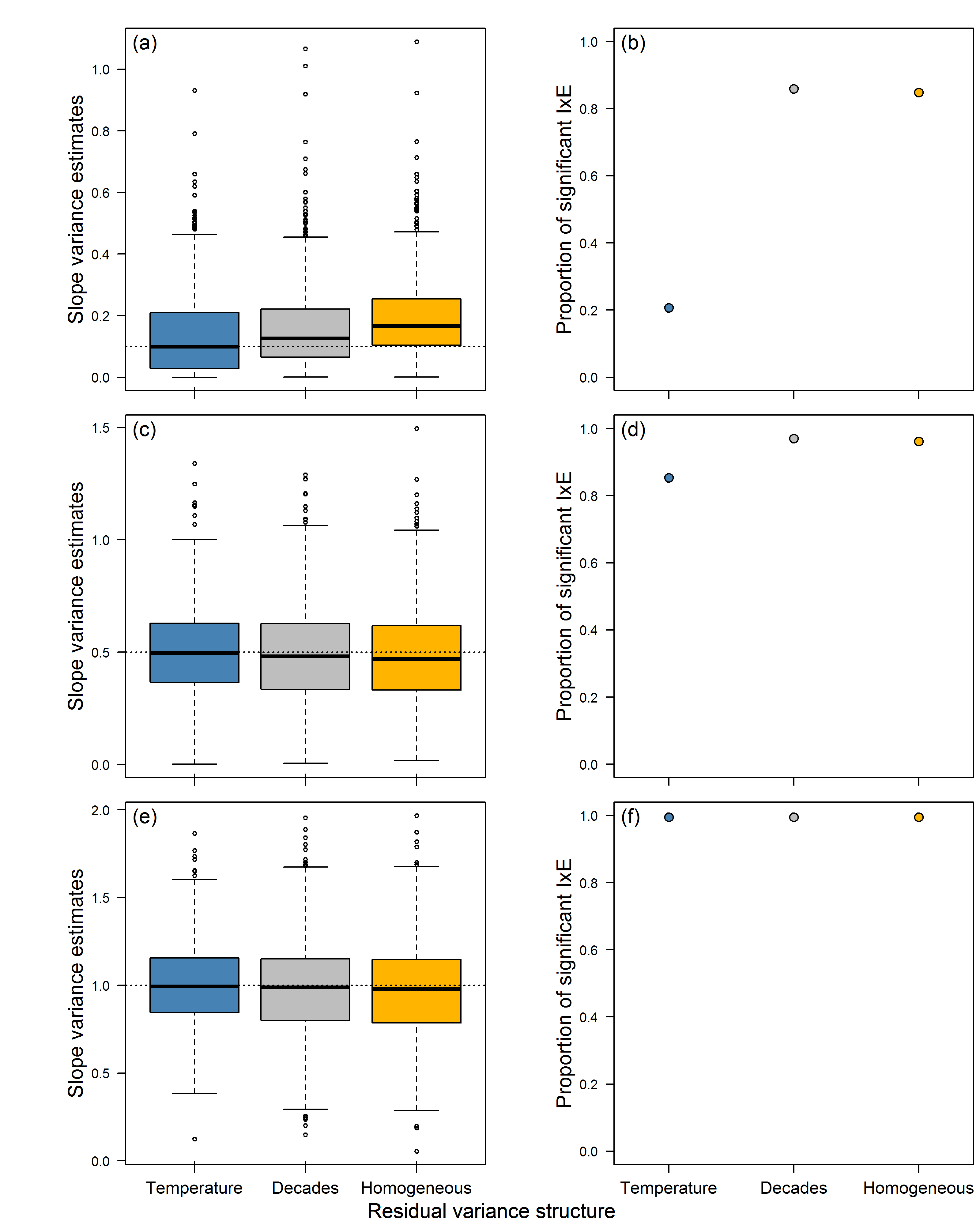
Figure S1.** Results of the simulation testing the effect of the residual structure of the random regression model on the estimate of variance in slopes (a, c, e) and the respective statistical power to detect this variance (b, d, f). Input values for slope variance are denoted by the horizontal, dotted lines.

**References**

Butler, D., Cullis, B.R., Gilmour, A.R. & Gogel, D.J. (2009) *ASReml–R Reference Manual, Release 3.0*. Department of Primary Industries and Fisheries, Brisbane, Qld, Australia.

Gienapp, P. & Brommer, J.E. (2014) Evolutionary dynamics in response to climate change. *Quantitative genetics in the wild* (eds A. Charmantier, D. Garant & L.E.B. Kruuk). Oxford University Press, Oxford, UK.

Gilmour, A.R., Gogel, B.J., Cullis, B.R. & Thompson, R. (2009) *ASReml User Guide. Release 3.0*. VSN International Ltd, Hemel Hempstead, UK.

Husby, A., Nussey, D.H., Visser, M.E., Wilson, A.J., Sheldon, B.C. & Kruuk, L.E.B. (2010) Contrasting patterns of phenotypic plasticity in reproductive traits in two great tit (*Parus major*) populations. *Evolution,* **64,** 2221–2237.

Husby, A., Visser, M.E. & Kruuk, L.E.B. (2011) Speeding up microevolution: the effects of increasing temperature on selection and genetic variance in a wild bird population. *Plos Biology,* **9,** e1000585.

Nussey, D.H., Postma, E., Gienapp, P. & Visser, M.E. (2005) Selection on heritable phenotypic plasticity in a wild bird population. *Science,* **310,** 304–306.
